# Magnetic resonance imaging texture analysis to differentiate ameloblastoma from odontogenic keratocyst

**DOI:** 10.1038/s41598-022-20802-7

**Published:** 2022-11-21

**Authors:** João Pedro Perez Gomes, Celso Massahiro Ogawa, Rafael V. Silveira, Gabriela Castellano, Catharina Simioni De Rosa, Clarissa Lin Yasuda, André Caroli Rocha, Bengt Hasseus, Kaan Orhan, Paulo Henrique Braz-Silva, Andre Luiz Ferreira Costa

**Affiliations:** 1 Department of Stomatology, Division of General Pathology, School of Dentistry, University of São Paulo (USP), São Paulo, SP Brazil; 2 Postgraduate Program in Dentistry, Cruzeiro Do Sul University (UNICSUL), São Paulo, SP Brazil; 3 Institute of Physics Gleb Wataghin, University of Campinas (UNICAMP), Campinas, SP Brazil; 4 Brazilian Institute of Neuroscience and Neurotechnology (BRAINN), Campinas, SP Brazil; 5 Department of Neurology, School of Medical Science, UNICAMP, Campinas, SP Brazil; 6 Division of Dentistry, Clinics Hospital of School of Medicine, USP, São Paulo, SP Brazil; 7 Department of Oral Medicine and Pathology, Institute of Odontology, The Sahlgrenska Academy, University of Gothenburg, Gothenburg, Sweden; 8 Department of Dentomaxillofacial Radiology, Faculty of Dentistry, Ankara University, Ankara, Turkey; 9 Laboratory of Virology, School of Medicine, Institute of Tropical Medicine of Sao Paulo, Universidade de São Paulo, Av. Prof. Lineu Prestes, 2227 – Cidade Universitária, São Paulo, SP Brasil

**Keywords:** Oral diseases, Magnetic resonance imaging

## Abstract

The differentiation between ameloblastoma (AB) and odontogenic keratocyst (OKC) is essential for the formulation of the surgical plan, especially considering the biological behavior of these two pathological entities. Therefore, developing means to increase the accuracy of the diagnostic process is extremely important for a safe treatment. The aim of this study was to use magnetic resonance imaging (MRI) based on texture analysis (TA) as an aid in differentiating AB from OKC. This study comprised 18 patients; eight patients with AB and ten with OKC. All diagnoses were determined through incisional biopsy and later through histological examination of the surgical specimen. MRI was performed using a 3 T scanner with a neurovascular coil according to a specific protocol. All images were exported to segmentation software in which the volume of interest (VOI) was determined by a radiologist, who was blind to the histopathological results. Next, the textural parameters were computed by using the MATLAB software. Spearman's correlation coefficient was used to assess the correlation between texture parameters and the selected variables. Differences in TA parameters were compared between AB and OKC by using the Mann–Whitney test. Mann–Whitney test showed a statistically significant difference between AB and OKC for the parameters entropy (*P* = 0.033) and sum average (*P* = 0.033). MRI texture analysis has the potential to discriminate between AB and OKC as a noninvasive method. MRI texture analysis can be an additional tool to differentiate ameloblastoma from odontogenic keratocyst.

## Introduction

The differentiation of ameloblastoma (AB) and odontogenic keratocyst (OKC) is essential for the formulation of a surgical plan, especially considering the particularities of these two pathological entities. Nonetheless, it is not a simple task. The results of differential diagnosis by imaging techniques are often unsatisfactory due to their radiological similarities^1,2^.

AB and OKC are both classified as benign odontogenic lesions, with the former presenting a considerably more locally aggressive behavior. Moreover, these lesions may occur in both jaws with higher prevalence in the body and posterior ramus of the mandible^1^.

Although similar from an imaging perspective, both lesions present significant differences regarding biological behavior, which is the reason why their differential diagnosis is of utmost importance in proposing a correct therapeutic approach. The recurrence rate of OKC may vary from 17 to 56% when the treatment is based on simple enucleation. However, if an adjunctive treatment is performed in order to complement the surgical therapy, such as decompression before enucleation or application of Carnoy´s solution, the recurrence rate is reported to be less than 2%^3^. With regard to OKC, the treatment based on complete resection may be considered unacceptable for some clinicians due to the benign nature of the lesion and the morbidity caused by this procedure^3^. In fact, it has been emphasized that the surgical approach of OKC must be determined by factors such as size of the lesion, patient age, cortical perforation, proximity to vital structures and recurrence of the lesion. Recent evidence demonstrated that decompression followed by enucleation has shown significant reduction of recurrence rate^4^.

AB, on the other hand, is a locally aggressive odontogenic neoplasm with recurrence rates reaching 90%^5^. Surgical clearance has been established as a key factor to reach an effective treatment. According to some authors, only complete resection can facilitate the cure^5^. Therefore, the chances of cure by using a conservative treatment are lower for AB in comparison to OKC.

Histological examination is currently essential for establishing an accurate diagnosis. Authors have emphasized that roentgenograms do not reveal the true nature of the lesion^6^.

Uniformity of the findings using imaging techniques has been described as a challenge for a correct diagnosis of OKC and AB ^2,7–9^. Moreover, there is consensus that such a diagnosis cannot be achieved without biopsy. Nevertheless, incisional biopsy usually consists in obtaining only small fragments of the lesions’ wall, which may not reflect their true nature as inflammation can mask the diagnostic features^10^.

Kitisubkanchana et al. have tried to use width-to-length ratio to differentiate AB from OKC, concluding that this method might be useful to help distinguish these lesions^10^. The authors compared the radiographic features of OKC and AB in conventional radiography and cone beam computed tomography for differential diagnosis of both lesions, but the correct diagnosis was found to be dependent on the experience of the radiologist.

Logistic regression analysis based on the presence of high-density areas has also been suggested as a possible way to differentiate AB from OKC. The results obtained by Ariji et al.^7^ showed that the presence of high-density areas could be used for differential diagnosis of the two lesions.

Texture analysis (TA) is a statistical imaging technique using radiomics to examine the distribution of the pixel signal intensity and the association between neighboring pixel values, thus allowing quantifying subtle differences in the image^11,12^. This enables one to perform a quantitative investigation of the image without the subjectivity of the human vision^13,14^.

In previous studies, TA has been applied to help distinguish between benign and malignant tumors in several locations of the human body, such as brain, rectum and lung ^15–18^. Oda et al.^19^ have shown the importance of this methodology in distinguishing cystic and cystic-appearing lesions by using computed tomography (CT) scan. However, this methodology has not yet been evaluated for discrimination between OKC and AB by means of magnetic resonance imaging (MRI).

Therefore, this study aimed to assess the values of TA parameters for differentiating OKC from AB in MRI scans.

## Materials and methods

### Subjects

The present prospective study was submitted to and authorized by the Research Ethics Committee of the Clinics Hospital of the University of São Paulo (USP) according to protocol number 90600718.5.0000.0068. All procedures in the study were performed in accordance with the human research ethical standards set by the local institutional board and the 1964 Helsinki Declaration, including later amendments. A written informed consent form was signed by all patients enrolled in the study. This study comprised 18 patients, all attending the Division of Dentistry of the Clinics Hospital from 2017 to 2020 for diagnostic and therapeutic purposes. Eight patients were diagnosed with AB and 10 with OKC. The inclusion criteria were the following: final histopathological diagnosis of AB or OKC and no clinical condition making the MRI exam unfeasible, such as presence of metallic prosthesis or claustrophobia. It is important to highlight that, with regard to the AB cases included, all of them were histologically classified as conventional ameloblastoma according to the 5th edition of the World Health Organization (WHO) Classification of Head and Neck Tumors ^20^. Therefore, the unicystic variant of AB was not included in the study.

Similarly, among the OKC cases included, all of them were histologically classified as odontogenic keratocyst as they are typically parakeratinized, in addition to being considered more aggressive and having a high recurrence rate^21^. Therefore, the orthokeratinized variant of OKC was not included in the study.

### Histopathological diagnosis

The histopathological diagnosis was performed by evaluating paraffin-embedded H&E stained tissue sections from incisional biopsies of lesions after MRI. The initial histopathological diagnosis was confirmed by analysing the surgically removed specimen after treatment. The diagnostic criteria followed the 5th edition of the World Health Organization Classification of Head and Neck Tumors: Odontogenic and Maxillofacial Bone Tumors^22^.

### Image acquisition

All patients were examined on a 3 T scanner (Achieva-Intera-Philips®) with a neurovascular coil. One sequence was included in the current study: axial T2-weighted imaging (Dixon Turbo Spin Echo) without fat suppression (time of repetition time [TR]/echo time [TE] = 3278/100 ms, voxel size of 0.79 × 1.15 × 4.00, FOV 200 × 263 × 158 mm and acquisition time of 1 min, 48 s). This sequence showed to be appropriate for revealing the most homogeneous regions of each lesion, an essential condition for segmentation and subsequent differentiation of intensity between neighboring pixels.

### Image processing and segmentation

All images were obtained in Digital Imaging and Communications in Medicine (DICOM) format and transferred to a computer (Dell Computer Corporation, Round Rock, TX, USA) with system 56DV4R2, 6 GB (RAM) and 32-inch monitor.

**Figure 1:**
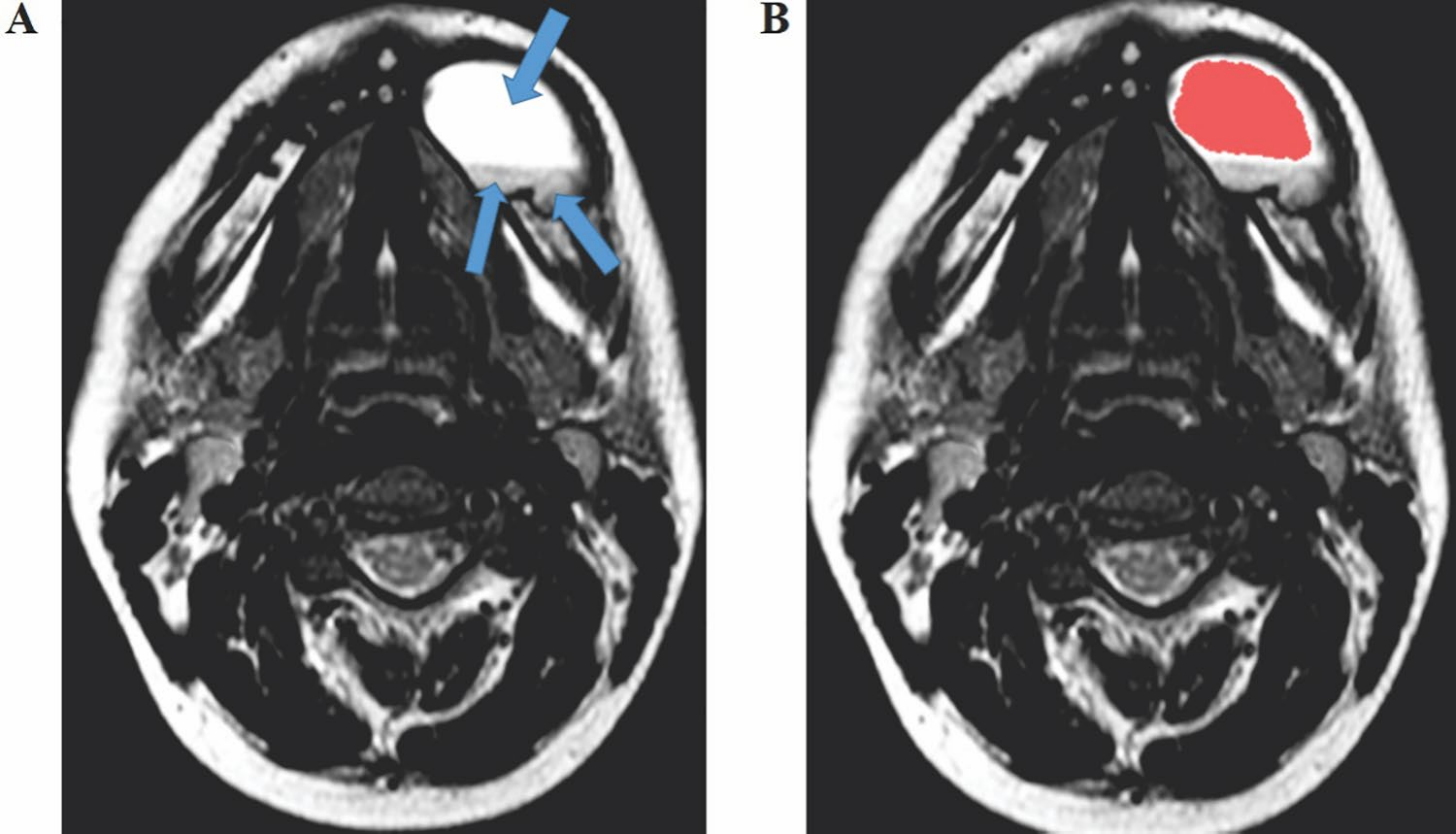
(**A**) Ameloblastoma located in the mandibular body on the left side. Arrows represent regions with variable signal intensity, which are characterized by hypersignal representing higher aqueous content, a characteristic of T2-weighted sequences. (**B**) Segmentation process represented by the red contour. Only the most homogeneous portion of the lesion was segmented, that is, the region shown by the hypersignal.

**Figure 2:**
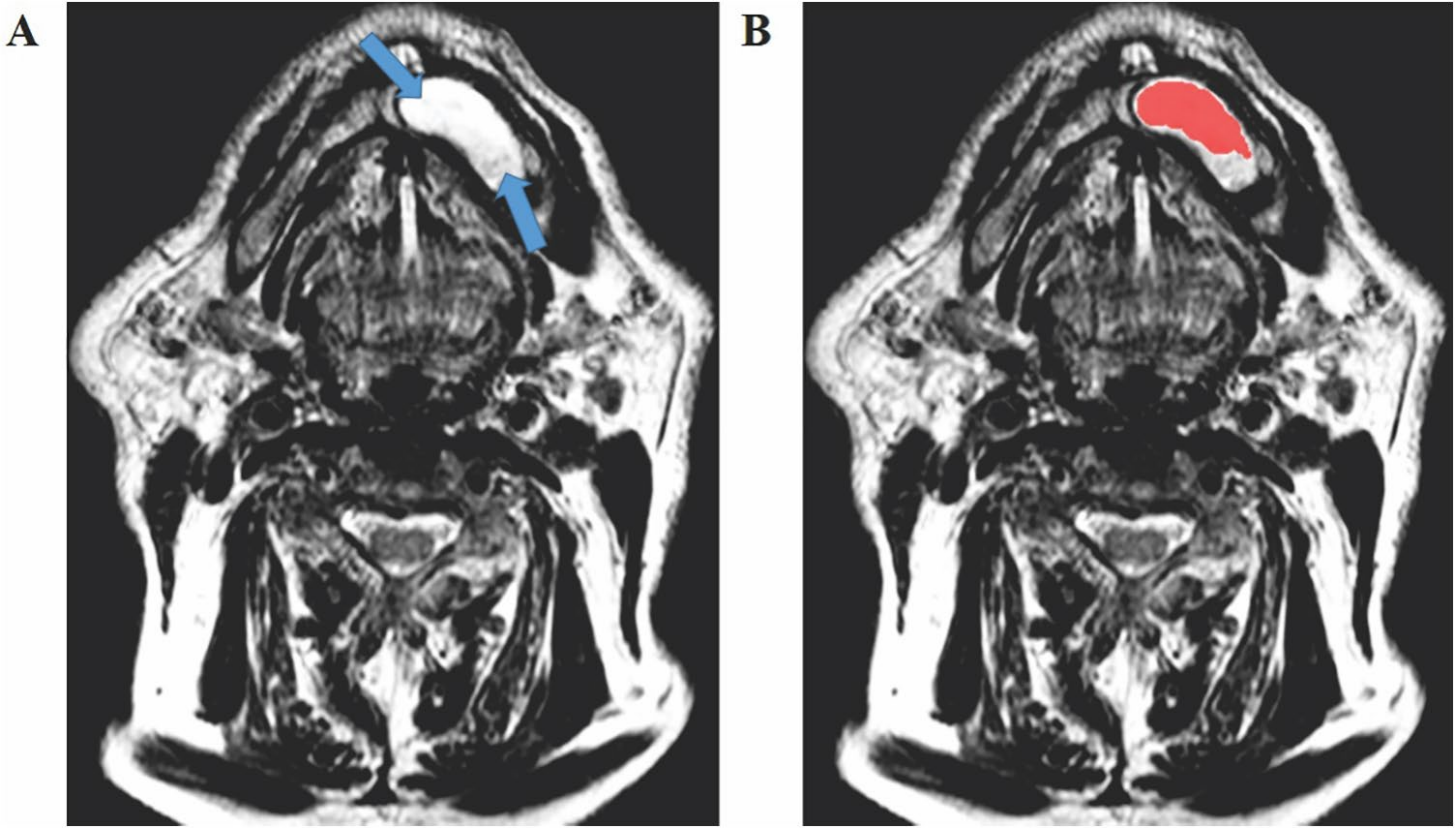
(**A**) Ameloblastoma with smaller volume compared to that shown in Fig. 1. However, the variable signal intensity along the entire length of the lesion is maintained, so that the most homogeneous region remains restricted to the portion characterized by the hypersignal, which is typical of T2-weighted sequences. (**B**) Segmentation process represented by the red contour. Only the most homogeneous portion of the lesion was segmented, that is, the region shown by the hypersignal.

MRI slices were chosen on the basis of optimal characterization of the largest lesion area. Two radiologists (with 5 and 15 years of experience in MRI) actively participated in the analysis of all cases. The segmentation was carried out manually with an agreement of the two radiologists. Image processing was initially performed by using the InVesalius® software (www.cti.gov.br/invesalius). Figures 1 and 2 demonstrate the segmentation process prior to determining the textural parameter values.

After segmentation of all lesions (i.e. VOI) and determination of their most homogeneous regions, the DICOM files were converted into NIfTI format by using the InVesalius® software toolbox.

### Parameters extraction

One radiologist performed TA as part of a blinded study by using home-made routines developed with MATLAB software (MathWorks, Natick, MA, USA). All segmented images were processed by using the MATLAB software (MathWorks, Natick, MA, USA) to extract the texture parameters.

Gray level co-occurrence matrix (GLCM) was the method used for TA, which is usually applied to two-dimensional images as it computes the co-occurrence of two gray-level values separated by a given distance for a given direction^23^. Here, GLCM was applied isotropically, in which all directions were investigated simultaneously and directly in three dimensions^24^. One-hundred and twenty-eight gray levels were used, as well as distances from 1 to 5 voxels, resulting in five 128 × 128 matrices for each VOI. A set of 11 Haralick parameters^23^ were extracted for each matrix: contrast, correlation, entropy of difference, entropy, homogeneity, sum average, sum entropy, sum variance, uniformity, variance of difference and variance (Table 1).

**Table 1:** Texture parameters used in the study.

Parameters	Description
Angular second moment	Measurement of the distribution (uniformity) of the gray-level image values. Images with low gray levels have more uniformity
Contrast	Amount of local variation in the gray levels. Higher levels of this parameter can indicate presence of edges, noises or streaks in the mage
Correlation	Measurement of the linear dependence of the gray levels between neighbor pixels, thus providing a measurement similar to that of self-correction methods
Sum of squares	Measurement of the dispersion of gray levels (variance) regarding the average
Inverse difference moment	Measurement of the smoothness (homogeneity) in the distribution of gray-level image values. If the value of contrast is low, then the inverse difference moment is high
Entropy	Measurement of the disorder degree between the pixels in the image, being the inverse of the angular second moment. Images with higher amount of gray levels have greater entropy
Sum of entropy	Measurement of the disorder degree related to the distribution of the sum of gray-level image values

The choice of voxel distances for GLCM (1 to 5) was made because the longer the distance between the co-occurring voxels, the larger has to be the VOI in order to have sufficient co-occurrence of voxel pairs. On the other hand, this size is limited by the size of the lesion. Also, given that we were looking for subtle tissue changes, we would expect them to occur at a not too long distance between the voxels. With regard to the number of gray levels, studies have demonstrated that a large number of gray levels implies in an increased shortage in GLCM. Therefore, we chose 128 levels, which were the optimal arrangement between shortage in GLCM and amount of information extracted. The Haralick parameters were also used in a previous study with good results^14,25,26^. It is important to mention that the number of slices used depended on the VOI, with GLCM being three-dimensionally calculated within the manually- segmented VOI.

### Statistical analysis

Spearman’s correlation coefficient test was used to determine the correlation between textural parameters and the distances between the neighboring pixels. AB and OKC differences were compared by using Mann–Whitney test. All analyses were performed by using the R software, version 3.6.0 (The R Foundation for Statistical Computing), at a significance level of 5%.

### Ethical approval

This study was approved by the Research Ethics Committee of the Clinics Hospital of the University of São Paulo (USP) according to protocol number 90600718.5.0000.0068.

### Informed consent

A written informed consent form was signed by all the patients enrolled in the study.

## Results

### Demographic and clinical characteristics of the patients

The demographic characteristics of the patients and features of the lesions are shown in Table 2. Of these 18 patients, 10 were male and eight were female, with 50% of the patients being white (n = 9). The lesions were located in the ramus of the mandible (n = 10), body of the mandible (n = 4) and maxilla (n = 4).

**Table 2:** Clinical aspects of patients and main features of the odontogenic keratocyst and ameloblastoma.

Variable	Mean ± SD	Median	Min–Max	N	Percentage
Age	35.5 ± 33.2	35.5	12–59	18	
**Gender**					
Male				10	55.6%
Female				8	44.6%
Total				18	100%
**Location**					
Ramus of the mandible				10	55.6%
Body of the mandible				4	22.6%
Maxilla				4	22.6%
Total				18	100%
**Skin color**					
White				9	50%
Not-white				9	50%
Total				18	100%
**Histopathological diagnosis**					
Odontogenic Keratocyst				10	55.6%
Ameloblastoma				8	44.6%

SD, Standard deviation; N, Sample size.

### Assessment of texture parameters

Eleven texture parameters were measured at five different distances (q1, q2, q3, q4 and q5), totaling 55 variables. In order to reduce the number of variables, the Spearman’s correlation coefficient test was used to determine the correlation between textural parameters and the respective distances.

**Figure 3:**
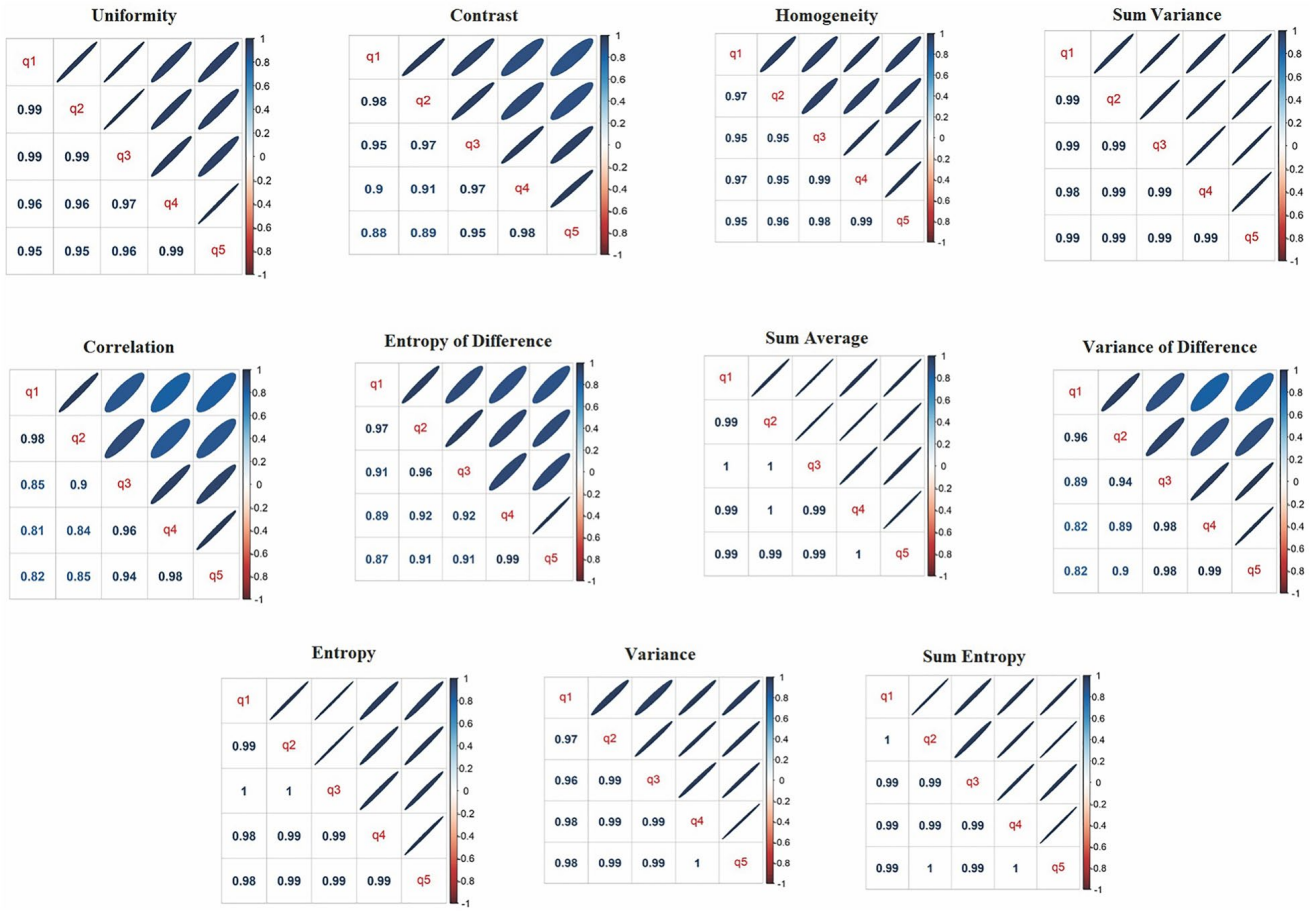
Correlation plots provide visual representation of the relationship between every texture parameters and the five positions based on Spearman's correlation coefficient. Narrower ellipses indicate stronger correlations. The distances were organized according to the five directions in the following positions: q1, q2, q3, q4 and q5.

A very strong correlation was found between the distances for four specific parameters: sum variance, sum entropy, variance of difference and entropy of difference. These four parameters showed a high correlation with at least one of the other seven remaining parameters, and for this reason, they were not considered in the following analyses. The exclusion of these four parameters was done in two steps:

1. The correlation of each parameter between the given directions was evaluated (Fig. 3). As the correlation is high, we can choose a single direction since the others provide the same information. Thus, q3 direction was used as reference for being the intermediate one.
2. In q3, the correlation between the parameters was assessed, and it was noticed that sum variance, sum entropy, variance of difference and entropy of difference presented a high correlation with at least one of the other remaining parameter. For this reason, they were excluded.

Figure 3 illustrates the correlation between the distances of each parameter evaluated in this study.

Once a strong correlation between q3 directions was determined and the variables restricted to seven parameters, the Mann–Whitney test was used to compare the texture parameters individually. Mann–Whitney test (Table 3) showed a statistically significant difference between the parameters entropy (*p*-value = 0.033) and sum average (*p*-value = 0.033) in comparison to the remaining parameters: uniformity, contrast, correlation, variance and homogeneity.

**Table 3:** Position and dispersion measurements of selected texture variables, by group and p-value of comparison between groups.

Parameter	Group	N	Mean	S.D	Average	*p*-value
Uniformity	AB	8	0.0013	0.0008	0.0014	0.286
	OK	10	0.0019	0.0010	0.0015	
Contrast	AB	8	522	351	426	0.051
	OK	10	241	149	242	
Correlation	AB	8	0.34	0.21	0.41	0.929
	OK	10	0.35	0.21	0.38	
Variance	AB	8	449	336	393	0.110
	OK	10	188	113	173	
Homogeneity	AB	8	0.15	0.04	0.17	0.929
	OK	10	0.15	0.05	0.14	
Entropy	AB	8	3.3	0.3	3.3	0.033
	OK	10	3.1	0.2	3.1	
Sum average	AB	8	104	43	101	0.033
	OK	10	63	21	57	

N, Sample size; S.D., Standard deviation; Q1 and Q3, Distances in which each variable (parameter) was measured.

**Figure 4:**
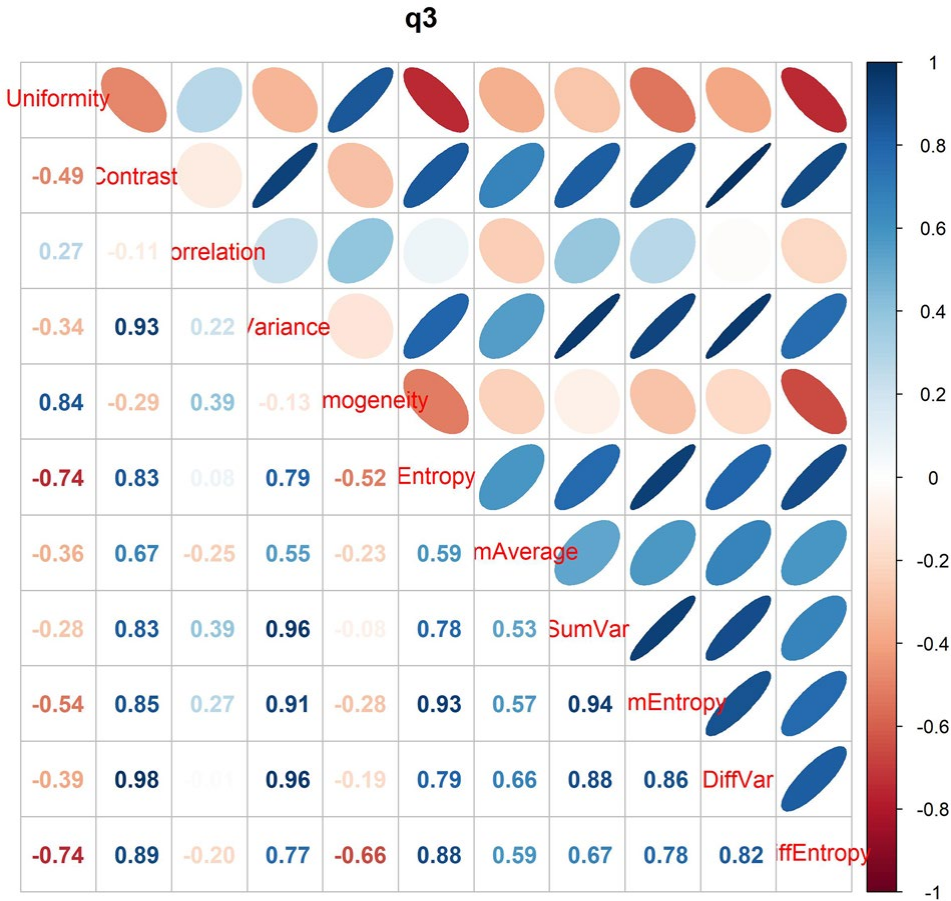
Correlation between variables within direction q3, chosen as reference for being the intermediate between all assessed directions. The high correlation of the textural parameters sum variance, sum entropy, variance of difference and entropy of difference with at least one remaining parameters is here represented.

Figure 4 shows the correlation between variables within direction q3, chosen for being the intermediate between the five evaluated directions. The high correlation of the four excluded parameters with at least one remaining parameters is represented in this figure.

## Discussion

Despite being radiologically similar, AB and OKC present a significantly different biological behavior with different recurrence rates, which is the reason why AB is treated less conservatively than OKC^4^. Developing means to allow discriminating one lesion from another by using imaging techniques, thus eliminating subjectivity, has been one of the greatest challenges in radiology field.

Artificial intelligence methods, specifically the machine-learning ones, have also been employed in an attempt to differentiate AB from OKC. Authors have proposed an algorithm with the intent to improve the accuracy of differential diagnosis of AB and OKC on panoramic radiographs^1^. Similarly, some authors have analysed the automatic classification performance of Google Inception v3, a convolutional neural network (CNN) using tomographic images of AB and OKC, concluding that a high classification accuracy was found in the images of AB despite the higher error rates^27^.

The present study has a similar premise. Like CNN, the application of MRI-based TA represents a possibility to increase the diagnostic power by combining imaging methods with gray-level recognition algorithms. This combination can provide us with important additional information to ensure accurate diagnosis and effective treatment with less morbidity. It has been emphasized that, although the diagnostic value of CNN models is considerably high, the application of this method is still not a reality due to a number of factors, such as computational costs and time-consuming segmentation steps^27^.

In these particular aspects, once the MRI protocol is properly standardized, MRI-based TA has advantages over other methods, as the segmentation can be performed with free software, thus making the whole process less expensive.

One of the principles guiding this study was the fact that cystic lesions of the jaw are clearly apparent on CT images and radiographs, however subtle differences in internal components within the lesions are often difficult to measure^19^. In this study, MRI texture analysis was performed in the most homogeneous regions of each lesion in order to prevent any eventual bias from selecting areas with different pixel intensities. This was done aiming to distinguish which of the analysed textural parameter presents statistical significant difference when both lesions are compared by having their internal region as the main reference with no distinguishable difference. We assumed that, as the OKC internal components are inherently different from those of AB, the textural features of both lesions should also be different.

It has been mentioned by other authors that the cystic spaces of OKCs are constituted by fluids with lower protein concentration^28^. Similarly, density differences related to the presence of desquamated keratin may help differentiate OKC from AB^19^.

There are some studies reporting the usefulness of diffusion-weighted MRI for diagnostic imaging of these two odontogenic lesions^29^. The authors concluded that the technique was able to discriminate AB from OKC on the basis of the presence of solid enhancing lesions. However, there are some limitations regarding this method, such as presence of desquamated keratinized materials, which may hamper the diffusion of OKC, and susceptibility to image artifacts as apparent diffusion coefficient values can change even with the use of the same MRI system^30,31^.

In this study, out of the 11 parameters extracted, two were found to have statistically significance evidence: entropy (*p*-value = 0.033) and sum average (*p*-value = 0.033), with the averages being lower for OKC. It is known that OKC has a lower density than AB^7^. Histologically, OKC has areas of desquamated keratin. The sum average textural parameter represents the average of sums of two-pixel values in the image of interest, whereas entropy shows the degree of disorder between pixels in the image. High classification accuracy was found in the images of AB despite the presence of keratin, which seems to give greater uniformity to OKC in the studied MRI sequence. Therefore, greater uniformity and lesser degree of gray-level disorder compared to AB. This suggests the existence of features associated with a MRI signal of greater uniformity in OKC than in AB.

Entropy is a parameter originating from the field of information theory and aimed at measuring the amount of disorder of a system^32^. In the case of texture analysis, entropy measures how random the gray-level distribution of voxels is. The sum average parameter is the mean of the distribution of the sum of co-occurring gray levels^32^. Authors have already demonstrated that for OKC, the average Hounsfield Unity (HU) density had lower values and higher heterogeneity^2^.The differences in sum average and entropy textural features found in this study may be related to the differences in density between AB and OKC.

In this study, we used the manual segmentation technique in which the radiologist outlines the lesion so that the computer algorithms can perform the analysis later. Although manual segmentation is more subjective, it is more accurate^33^, even more considering that we used two radiologists in this step. Our results could assist the surgeon in exploring the lesion non-invasively, that is, with no need of biopsy. Additionally, we used public free software so there should not be any additional cost to the patient.

We demonstrated that extracted features from lesions on MRI images can be used for characterization of AB and OKC as well as for diagnostic supplementation.

This study, however, has several limitations which should be acknowledged. Firstly, the relatively small sample. Secondly, the sample had no case of unicystic ameloblastoma and orthokeratinized odontogenic keratocyst. Therefore, further studies on other histological types should be performed for comparisons with texture parameters. As for the relatively small sample, it is important to highlight that because TA is based on the signal intensity of pairs of pixels, the sample size is not only related to the number of patients, but mainly to the volume of the lesions. Thus, the higher the volume is, the larger the region of segmentation and the greater the amount of pixels in each image. At last, drawing the VOIs manually may have led to a certain amount of variability.

As the perspectives for the continuation of this study rely on the automation of the segmentation process and statistical analysis of the images, it is essential to make this tool easy and fast so that it can be used routinely.

## Conclusions

Our results show that entropy and sum average are textural parameters of T2-weighted images which can be used without fat suppression for diagnosis of radiologically similar lesions, such as AB and OKC. Therefore, MRI texture parameters are a sensitive and efficient method to detect both lesions and could be of high value to assist in the therapeutic decision-making process.

## Data Availability

The datasets and/or analysed during the current study are available from the corresponding author on reasonable request.
